# Workshop Characteristics Related to Chronic Disease Self-Management Education Program Attendance

**DOI:** 10.3389/fpubh.2015.00019

**Published:** 2015-04-27

**Authors:** Matthew Lee Smith, Marcia G. Ory, Luohua Jiang, Kate Lorig, Kristie P. Kulinski, SangNam Ahn

**Affiliations:** ^1^Department of Health Promotion and Behavior, College of Public Health, The University of Georgia, Athens, GA, USA; ^2^Department of Health Promotion and Community Health Sciences, Texas A&M Health Science Center School of Public Health, College Station, TX, USA; ^3^Department of Epidemiology, School of Medicine, University of California Irvine, Irvine, CA, USA; ^4^Stanford Patient Education Research Center, Department of Medicine, Stanford University School of Medicine, Palo Alto, CA, USA; ^5^National Council on Aging, Washington, DC, USA; ^6^Division of Health Systems Management and Policy, School of Public Health, The University of Memphis, Memphis, TN, USA

**Keywords:** chronic disease self-management, evidence-based program, older adults, intervention dose, evaluation

## Abstract

Using the national dissemination of Chronic Disease Self-Management Education (CDSME) programs, the purposes of this study were to (1) document intervention attendance rates as related to the number of participants enrolled in the workshop and (2) compare the relationship between workshop attendance and workshop size by delivery site rurality and type. Data were analyzed from the first 100,000 middle-aged and older adults who participated in CDSME workshops spanning 45 states, Puerto Rico, and the District of Columbia as part of the American Recovery and Reinvestment Act of 2009 *Communities Putting Prevention to Work: Chronic Disease Self-Management Program* initiative. Descriptive statistics are reported for all participants, then separately by each delivery site type. Ratios between the number of workshop participants and the number of workshop sessions attended were calculated and graphed based on the rurality of delivery and separately for the leading five delivery site types. Associations between the number of workshop participants and the number of sessions attended differed by delivery site rurality and type. Findings have implications for participant retention and workshop delivery costs, which can assist program deliverers to strategically plan implementation efforts in their areas.

## Introduction

The recent movement toward evidence-based public health calls for a better understanding of the implementation and dissemination of evidence-based programs (EBP) for older adults delivered in real world settings (1–4). EBP are interventions based on research that were tested in clinical trials and translated into community-based models, which receive the same intended health benefits (5). EBP have common components, foremost of which are essential intervention elements, materials, and procedures (6). More specifically, implementation features must be considered, which include having a well-defined program structure and timeframe that enables the developers to track fidelity and others to uniformly deliver the program with replicable findings (7). Program developers often draw upon small group literature and adult learning principles to define the ideal class size for intervention (8–10), which is often 12–16 participants (11). From our experience working with program developers, determination of ideal class size is often more of an art than a science and is based on assumptions about ideal size to facilitate active group discussion. Secondary concerns often revolve around cost implications of different class sizes in intervention studies because per-participant costs are influenced by the total number of participants enrolled in workshops (12, 13).

With a desire to take EBP to scale in order to make a public health impact, there is a need for widespread penetration in the designated population of interest (14, 15). To counter recruitment challenges often seen in research studies (16–18), there is now a growing literature on strategies to increase recruitment by facilitating program adoption in a host of different delivery systems reflecting where the population of interest resides and frequently encounter in their daily lives.

Despite the assumed importance of structured program features such as class size or workshop delivery type, little is known about the programmatic impact of different delivery characteristics on achieving recommended intervention doses. This is, in part, because assumptions about ideal class size are often applied from prior literature without consideration of the specific intervention focus, population, or setting. Delivery sites may be seen as implementation issues rather than researchable variables, an attitude reinforced by the limited number of delivery site types included in most intervention studies.

The widespread availability of Chronic Disease Self-Management Education (CDSME) programs nationwide across a multitude of settings has provided opportunity to examine the programmatic impact of different delivery characteristics on participants receiving the recommended intervention dose. Using the national dissemination of CDSME programs, the purposes of this study were to (1) document intervention attendance rates as related to the number of participants enrolled in the workshop and (2) compare the relationship between workshop attendance and workshop size by delivery site rurality (i.e., metro, non-metro) and type (e.g., senior centers, healthcare organizations, residential facilities, faith-based organizations).

## Materials and Methods

### Program description

The Chronic Disease Self-Management Program (CDSMP) has been introduced and widely disseminated in the U.S. as a method to empower patients with self-management skills to deal with their chronic conditions (19). There is now a suite of CDSME programs licensed through the Stanford Patient Education Research Center, some of which are generic (e.g., CDSMP, Tomando Control de su Salud) and others that are disease specific (e.g., diabetes, arthritis, chronic pain). Drawing upon Social Learning Theory (20), CDSME programs are evidence-based, peer-led interventions consisting of six highly participative classes held for 2.5 h each, once a week, for six consecutive weeks (19). CDSME programs have resulted in improved health care and health (21, 22), while potentially saving healthcare costs (12).

### Data source and study population

Cross-sectional data for this study were obtained from a nationwide delivery of CDSME programs as part of the American Recovery and Reinvestment Act of 2009 (i.e., ARRA) *Communities Putting Prevention to Work: Chronic Disease Self-Management Program* initiative (15). The US Administration on Aging led this initiative in collaboration with the Centers for Disease Control and Prevention and the Centers for Medicare and Medicaid Services to support the translation of CDSME programs in 45 states, Puerto Rico, and the District of Columbia (23). Federal funding for this initiative enabled participants to enroll in CDSMP workshops free of charge. This initiative was originally designed to have 50,000 Americans complete at least four out of six CDSME workshop sessions between 2010 and 2012 and to embed CDSME program delivery structures into statewide systems (15). For this study, data were analyzed from the first 100,000 participants who attended CDSME program workshops and responded to all relevant survey questions. Workshops included in study analyses began between January 2010 and February 2012.

As described elsewhere (24), all states receiving ARRA funding for this initiative were assigned program completer target goals. It was expected that CDSME program workshops would be delivered through certain site (e.g., senior centers, healthcare organizations, residential facilities, educational institutions, faith-based organizations, and tribal centers). Each delivery site type recruited participants to enroll in workshops using their usual methods (e.g., flyers, emails, face-to-face). The majority of participants was introduced to the program during the first workshop session; however, some participants were introduced to the program during an optional pre-workshop session called a “session zero” (25).

### Measures

#### Workshop attendance

Participants’ attendance was recorded to determine if the recommended intervention dose was received. As defined by the program developers, a participant has “successfully” completed the program if they attended four or more of the six offered workshop sessions (15, 21, 22).

#### Class size

The number of participants enrolled in each CDSME workshop was recorded (i.e., ranging from 1 to 20 participants). The maximum number of participants allowed to be listed as enrollees in any single workshop was 20. As a point of reference, the program developers define the ideal class size as (i.e., between 10 and 15 participants) in the CDSMP fidelity manual (26).

#### Delivery site types

Data are presented for all 10 delivery site types (see Table 1), which were then assessed graphically based on the workshop rurality and independently for the leading five delivery site types based on participant enrollment (i.e., senior centers, healthcare organizations, residential facilities, community/multi-purpose facilities, and faith-based organizations). Data pertaining to CDSME program delivery site types were gathered administratively (24). Using the ZIP code information provided by each delivery site, workshops were categorized as metro or non-metro based on the rural–urban commuting area codes (RUCA) (27). The leading five CDSMP delivery site types included in analyses were senior centers or area agencies on aging (AAAs), healthcare organizations, residential facilities, community or multi-purpose centers (including libraries), and faith-based organizations.

**Table 1:** **Participant and workshop characteristics by delivery site type**.

	Total (*n* = 100,000)	Senior Center/AAA (*n* = 29,152)	Healthcare Organization (*n* = 21,136)	Residential Facility (*n* = 17,631)	Comm/Multi-Purp/Library (*n* = 9,891)	Faith-Based Organization (*n* = 8406)	Educational Institution (*n* = 2264)	Health Department (*n* = 1274)	Tribal Center (*n* = 189)	Workplace (*n* = 541)	Other (*n* = 9,516)
**PARTICIPANT CHARACTERISTICS**											
Age	67.1 (±14.58)	71.1 (±11.76)	61.9 (±14.49)	73.5 (±12.96)	65.7 (±14.01)	65.7 (±13.71)	61.8 (±15.85)	64.2 (±14.59)	64.6 (±13.46)	60.8 (±14.80)	59.0 (±17.21)
Female	77.9%	80.5%	73.3%	82.7%	78.2%	78.9%	82.8%	79.6%	72.0%	81.7%	68.8%
Race/Ethnicity
Latino/Hispanic	16.6%	12.4%	27.3%	10.4%	17.1%	13.0%	27.7%	7.7%	5.1%	5.4%	21.2%
Non-Hispanic White	54.4%	58.0%	51.7%	60.0%	49.9%	44.1%	58.6%	75.0%	24.3%	63.3%	48.6%
African-American	21.8%	23.6%	14.6%	23.7%	20.8%	34.0%	10.6%	9.4%	7.3%	13.5%	22.0%
Asian/Pacific Islander	4.6%	3.6%	3.2%	3.6%	10.1%	7.1%	2.3%	4.8%	0.0%	14.4%	4.2%
American Indian/Alaska native	1.5%	1.2%	1.9%	1.0%	0.9%	0.8%	0.4%	1.5%	61.0%	1.7%	3.0%
Other race	1.2%	1.1%	1.3%	1.4%	1.1%	1.1%	0.6%	1.6%	2.3%	1.7%	1.0%
Average number of co-morbidities	2.2 (±1.71)	2.3 (±1.71)	2.1 (±1.68)	2.4 (±1.80)	2.1 (±1.70)	2.0 (±1.60)	2.0 (±1.66)	2.2 (±1.78)	2.7 (±1.80)	1.9 (±1.59)	2.0 (±1.63)

**WORKSHOP CHARACTERISTICS**											
Class size	12.7 (±4.18)	13.1 (±4.26)	12.1 (±4.13)	13.1 (±4.01)	12.5 (±4.18)	12.9 (±4.09)	12.9 (±4.10)	10.3 (±4.28)	9.6 (±2.74)	10.7 (±3.89)	12.8 (±4.12)
Number of sessions attended	4.4 (±1.72)	4.5 (±1.65)	4.2 (±1.78)	4.2 (±1.81)	4.4 (±1.70)	4.5 (±1.63)	4.5 (±1.70)	4.2 (±1.75)	4.2 (±1.69)	4.7 (±1.52)	4.7 (±1.66)
Successful completion	74.9%	77.0%	72.1%	70.2%	74.7%	78.7%	77.3%	69.2%	69.3%	82.6%	79.7%
Proportion of workshops in non-metro areas	20.4%	22.4%	17.7%	16.4%	17.1%	22.2%	25.8%	36.7%	13.8%	31.8%	25.7%

#### Personal characteristics

Personal characteristics of the participants included age, gender, race/ethnicity, and self-reported number of chronic conditions (i.e., arthritis, cancer, depression, diabetes, heart disease, hypertension, lung disease, stroke, osteoporosis, and other chronic conditions).

### Analyses

Descriptive statistics were calculated for all participants, then separately for each of the 10 delivery site types. Percentages are provided for categorical variables. Averages and standard deviations are provided for continuous and count variables. The average number of workshop sessions attended by the size of the workshop (i.e., the number of participants enrolled in each workshop) was calculated and graphed based on the rurality of delivery and separately for the leading five delivery site types.

## Results

### CDSMP participant and workshop characteristics by delivery site type

Of the first 100,000 participants reached in this initiative, 29.2% attended workshops at senior centers/AAAs, 21.1% at healthcare organizations, 17.6% at residential facilities, 9.9% at community/multi-purpose facilities (including libraries), and 8.4% at faith-based organizations. Smaller proportions of participants attended workshops at educational institutions (2.3%), county health departments (1.3%), workplaces (0.5%), and tribal centers (0.2%). Approximately 9.5% of participants attended CDSME workshops at delivery sites classified as “other” (e.g., correctional facilities malls, RV parks, fire departments, county administration buildings, private residences, casinos, career centers).

On average, participants were age 67.1 (±14.6) years and had 2.2 (±1.7) self-reported chronic conditions. The majority of participants were female (77.9%) and non-Hispanic white (54.4%). Almost 22% of participants were African-American and 16.6% were Hispanic. Workshops at senior centers/AAAs and residential facilities enrolled participants with older than average ages. Healthcare organizations, tribal centers, and sites categorized as “other” enrolled larger proportions of male participants. Healthcare organizations, community/multi-purpose facilities, educational institutions, and other sites enrolled larger proportions of Hispanic participants. Senior centers/AAAs, residential facilities, and faith-based organizations enrolled larger proportions of African-American participants. Healthcare organizations, residential facilities, community/multi-purpose facilities, and tribal centers enrolled more participants from metro areas. Workshops at senior centers/AAAs, residential facilities, and tribal centers enrolled participants with higher than average co-morbidities.

More than 20% of participants attended workshops delivered in non-metro areas. The workshops in non-metro areas had smaller average class size than those in metro areas, but no difference in class attendance. On average, workshops had 12.7 (±4.2) participants, and participants attended an average of 4.4 (±1.7) of the six workshop sessions. The majority of participants successfully completed the workshop (74.9%), indicating they received the recommended intervention dose. Senior centers/AAAs, faith-based organizations, educational institutions, and delivery sites categorized as “other” had higher than average workshop sizes and workshop attendance. Residential facilities also had higher than average workshop sizes. Workplaces also had higher than average workshop attendance.

### Associations between workshop size and attendance

**Figure 1:**
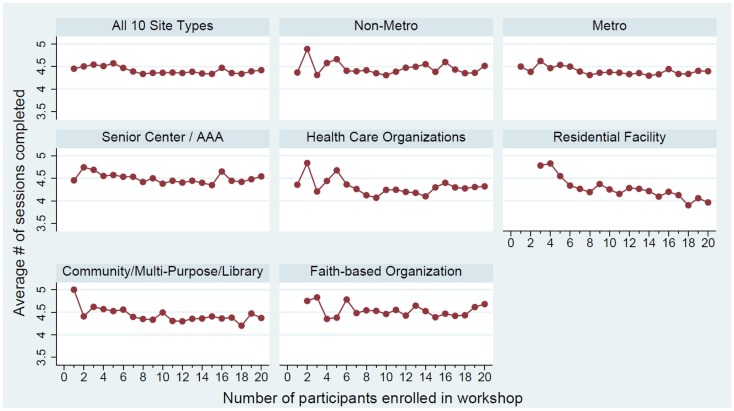
**Average number of workshop sessions attended by class size by workshop rurality and leading five delivery site type**.

As shown in Figure 1, associations between the number of workshop participants and the number of sessions attended differ by workshop rurality. More specifically, in workshops in metro areas, there was a negative correlation between participant number and session attendance for smaller workshops (i.e., workshops ≤ 8 participants). The average number of sessions attended in non-metro organizations had higher variability, especially for smaller workshops. Associations between the number of workshop participants and the number of sessions attended differed by delivery site type. More specifically, in senior centers/AAAs, there was a negative correlation between participant number and session attendance for smaller workshops (i.e., workshops ≤ 8 participants). Stated differently, the fewer the participants enrolled in a workshop, the higher the rate of session completion. For workshops with nine or more participants, the workshop size was not correlated with the average number of sessions attended. The relationships between participant number and session attendance in healthcare organizations and community/multi-purpose/libraries were similar to that observed in senior centers/AAAs. However, the average number of sessions attended in healthcare organizations had higher variability, especially for smaller workshops.

For delivery sites located in residential facilities, the number of workshop participants was negatively associated with session attendance. Generally, workshops with fewer participants had higher average workshop attendance. In faith-based organizations, however, there was no observed association between the number of workshop participants and workshop attendance.

## Discussion

As demonstrated in a previous study, workshop size was associated with workshop attendance in national dissemination efforts of EBP for older adults (13). As confirmed by other studies (15, 22), most delivery sites reported workshop sizes in the ideal range (i.e., between 10 and 15 participants) and large proportions of participants with successful completion rates. While general findings in the current study indicate that workshops with fewer participants had higher attendance rates, variability was noted by setting, especially among smaller workshops. Greater variability in smaller workshop as observed in workshops delivered in non-metro areas, healthcare organizations, and faith-based organizations. The strongest negative association was observed in residential facilities.

Consistent with the RE-AIM planning and evaluation framework (28, 29), wide-scale programmatic dissemination to diverse population subgroups often requires a multitude of community partnerships representing various settings. It is not surprising that senior centers/AAAs and healthcare organizations serve as the predominant sites, given the sponsorship of this initiative by the Administration on Aging (23). However, it is more interesting to consider the different delivery settings utilized in this initiative’s program implementation and dissemination activities (e.g., senior centers, healthcare organizations, residential facilities, faith-based organizations). This study contributes to the emerging implementation science literature by identifying and documenting the wide variability in workshop size and attendance based on different setting types (30).

As seen elsewhere (31, 32), certain delivery site types are known to attract participants with certain characteristics, which make it difficult to disentangle the impact of workshop size and attendance from the types of participants who attend a particular delivery site type. Future research would benefit from qualitative research to better understand what drives participants to one setting or another. For example, is participant attendance related to the supply of programing at different settings? Is it that participants identify with a particular organizational setting and, therefore, attend workshops where they are most comfortable (33)? Or, is it simply a proximity issue in that participants attend workshops that are closes to their home or work (34)? Or, might it be a combination where participants are willing to travel further distances to attend workshops delivered in a setting of preference? These issues warrant further investigation at the individual-level based on preference and the workshop-level based on common characteristics associated with workshop size and attendance.

These study findings are illuminating in that they show the interconnectedness of and interaction between workshop size, delivery site type, and intervention dosage. Findings indicate that there is no “one size fits all” rule of thumb regarding ideal workshop size and that the recommended intervention dose can be obtained at different delivery settings in workshop of differing sizes. Additional research is needed to determine the influence of workshop size and attendance on known health-related benefits associated with CDSME programs, controlling for workshop delivery site. Further, there is need for more sophisticated threshold analyses to determine the critical class size for optimal attendance and how that may differ by delivery site type.

Several study limitations can be noted. First, the cross-sectional nature of the study and lack of outcome data limited our ability to determine causality and associate workshop size and attendance with salient health outcomes. Second, there were a limited number of variables collected about the delivery sites and/or from participants; thus, we were unable to investigate the greater context of factors related to delivery site type selection, reasons for attendance, or reasons why certain delivery sites held workshops of certain sizes. However, the large number of workshops delivered and participants enrolled in this national initiative provides an initial glimpse into study questions and suggests areas needing more exploration. Third, this descriptive study was served as a preliminary examination of the relationship between workshop characteristics (delivery site type and number of participants) and class attendance. Future studies with more sophisticated, inferential statistics that include more predictor variables are needed to compare these relationships by other factors (e.g., self-reported chronic condition types) and health-related improvements resulting from intervention attendance.

This research has several practical implications. First, multi-pronged strategies are needed to improve participant retention so participants can receive the recommended intervention dose, despite workshop enrollment size. These strategies should be tailored approaches by delivery site types based on their specific participant characteristics and health-related status (35). Second, while class size may not always be associated with intervention dose, class size has implications for overall program costs (12, 36). This has been seen in our calculations of the cost savings that can be derived from CDSME programs based on variations in overall per-participant costs, which is highly dependent upon class size (12, 36). More specifically, based on a workshop cost of $3500 USD, per-participant costs can range from about $219 USD for larger workshops with 16 participants to $583 USD for smaller workshops with 6 participants (12). As such, because CDSME program workshops have a fair amount of fixed costs, regardless of workshop size (e.g., associated with site coordination, participant recruitment), hosting larger versus smaller workshops seems to be more fiscally beneficial to organizations implementing these programs. These cost-related variations have implications for program administrator and decision makers to finically plan future dissemination efforts and identify necessary resources and partners to achieve participant recruitment goals. Further, because of its small group approach using the social learning theory (19, 20), workshop size should be considered to ensure the program operates as intended and participants receive anticipated intervention benefits.

## Conclusion

The implementation processes in a national rollout of evidence-based CDSME programs are necessarily complex. Previous assumptions about the ideal class size need to be weighed in terms of both programmatic and cost metrics, balancing the economies of “going to scale” with the benefits of smaller class sizes in some settings. Therefore, it is important to recognize how delivery sites cater to different types of participants, which may in turn influence program outcomes. Findings have implications for participant retention and workshop delivery costs, which can assist program deliverers to strategically plan implementation efforts in their areas.

## Conflict of Interest Statement

The authors declare that the research was conducted in the absence of any commercial or financial relationships that could be construed as a potential conflict of interest.

This paper is included in the Research Topic, “Evidence-Based Programming for Older Adults.” This Research Topic received partial funding from multiple government and private organizations/agencies; however, the views, findings, and conclusions in these articles are those of the authors and do not necessarily represent the official position of these organizations/agencies. All papers published in the Research Topic received peer review from members of the Frontiers in Public Health (Public Health Education and Promotion section) panel of Review Editors. Because this Research Topic represents work closely associated with a nationwide evidence-based movement in the US, many of the authors and/or Review Editors may have worked together previously in some fashion. Review Editors were purposively selected based on their expertise with evaluation and/or evidence-based programming for older adults. Review Editors were independent of named authors on any given article published in this volume.
